# The workplace culture, mental health and wellbeing of early- and mid-career health academics: a cross-sectional analysis

**DOI:** 10.1186/s12889-024-18556-0

**Published:** 2024-04-23

**Authors:** Claudia H. Marck, Darshini Ayton, Trevor Steward, Hui-Fern Koay, Joshua F. Wiley, George Taiaroa, Courtney C. Walton, Isabelle Weld-Blundell, Matthew D. Greaves, Ankur Singh

**Affiliations:** 1 Disability and Health Unit, Melbourne School of Population and Global Health, The University of Melbourne, Parkville, VIC 3010 Australia; 2 Health and Social Care Unit, School of Public Health and Preventive Medicine, Monash University, 553 St Kilda Rd, Melbourne, VIC 3004 Australia; 3 Melbourne School of Psychological Sciences, University of Melbourne, Parkville, VIC 3010 Australia; 4 Melbourne Neuropsychiatry Centre, Department of Psychiatry, The University of Melbourne, Parkville, VIC 3010 Australia; 5 Department of Microbiology and Immunology, Peter Doherty Institute for Infection and Immunity, University of Melbourne, Parkville, VIC 3010 Australia; 6 Turner Institute for Brain and Mental Health and School of Psychological Sciences, Monash University, 18 Innovation Walk, Clayton, VIC 3800 Australia; 7 Department of Infectious Diseases, The University of Melbourne at the Peter Doherty Institute for Infection and Immunity, Parkville, VIC 3010 Australia; 8 Victorian Infectious Diseases Reference Laboratory, The Royal Melbourne Hospital at The Peter Doherty Institute for Infection and Immunity, Parkville, VIC 3010 Australia; 9 Centre for Epidemiology and Biostatistics, Melbourne School of Population and Global Health, University of Melbourne, Parkville, VIC 3010 Australia; 10 Melbourne Dental School, University of Melbourne, Parkville, VIC 3010 Australia; 11 The Melbourne School of Population and Global Health, The University of Melbourne, Parkville, VIC Australia

**Keywords:** Workplace culture, Academia, Workplace abuse, Burnout, Mental health

## Abstract

There are reports of poor working conditions for early and mid-career academics (EMCAs) in universities, however, empirical data using validated tools are scarce. We conducted an online, cross-sectional survey using validated tools to assess workplace satisfaction, exposure to workplace abuse, and mental health. Participants included employees of medical and health faculties of two of the largest Australian universities, surveyed between October 2020 and January 2021.

Overall, 284 participants responded. Many reported job insecurity: half (50.7%) working on contracts with less than one remaining year. Workloads were considerable, with 89.5% of participants working overtime and 54.8% reporting burnout. Workplace abuse in the forms of bullying (46.6%), sexual harassment (25.3%), sexism (49.8%) and racism (22.5%) were commonly reported. Clinically significant symptoms of depression (28.0%), anxiety (21.7%) and suicidal ideation or self-harm (13.6%) were reported; with a higher prevalence among those working more overtime, and those exposed to workplace abuse. Priorities include providing a stable and safe workplace, increasing accountability and transparency in addressing workplace abuse, and supporting professional development.

In summary, EMCAs in our study were commonly exposed to precarious employment conditions and workplace abuse. Our findings provide empirical evidence on where universities and funding bodies should direct resources and change organisational risk factors, to improve workplace culture.

**Supplementary Information:**

The online version contains supplementary material available at 10.1186/s12889-024-18556-0.

## Introduction

Employment conditions are a critical social determinant of health and health inequities [1–3]. Precarious employment and a stressful work environment have long-term damaging health impacts [1]. The academic workforce in health, medical and biomedical research (henceforth health academics for brevity) provides a pivotal role in all societies. They train and prepare the future health research workforce and undertake a diverse range of research to save lives and improve individual and population health. Yet, it is reported that health academics operate within a challenging system [4]. This context has been characterised as competitive, demanding, and unstable [3], with funding pressures, difficulty maintaining work life balance, and experiences of abuse commonly reported [5–7]. Reports of exposure to workplace abuse, characterised by bullying, harassment, and discrimination, contribute to a toxic and stressful work environment [8, 9]. Many exhibit low job satisfaction, experience stressful working conditions, and consider leaving academia [7, 8, 10, 11].

Precarious employment conditions are heightened at the early and mid-career academic (EMCA) stage, where staff experience a major employment bottleneck due to the limited and competitive funding structure of medical and health research in Australia. Globally, Australian universities are well-regarded in terms of providing high quality training for health academics, but research on Australian health academics wellbeing has been limited. Within Australia, EMCAs in all fields face a high degree of casual and temporary employment leading to job insecurity and associated stress [8, 10]. While casual employment traditionally is considered a pathway for engaging clinicians in academic teaching and research, over-casualisation and temporary fixed-term employment has increasingly become the norm within health and medical institutes [12, 13], due to the significant reliance on, and tightening of, research grant funding. Consequently, opportunities for tenured academic roles are sparse making the Australian health academics uniquely exposed to mental health stressors [7].

The COVID-19 pandemic has had a significant impact on mental health of people [14]. Impacts of the pandemic on mental health of academics from health/medical facilities were potentially even higher [15, 16], because in addition to suffering the impacts of disruptions to society, economy and increased caring responsibilities; this group has also been at the forefront as clinicians, nurses, public health researchers and biomedicine experts addressing urgent public needs. This additional set of stressors coupled with increased research and teaching workloads, office and laboratory closures and adapting to online working environments makes academics from medical and health faculties a unique cohort with mental health concerns. The literature also suggests that some subgroups within academia, for example women or EMCAs, were more impacted by the pandemic [16, 17].

There is a paucity of robust data about EMCA experiences of their workplace and their health and wellbeing and work satisfaction at this crucial part of their careers [18]. There are three key evidence gaps that this study will address. First, previous surveys on the working conditions of EMCAs from medical and health faculties have rarely applied validated tools for measuring work characteristics or mental health outcomes [8, 10, 19, 20]. This study aimed to use validated tools enabling comparisons between the general population, other work environments to examine if working conditions and mental health is similar or worse between population groups. Second, working conditions may differ between faculties within a university context. For example, the funding landscape for health academics is very different compared to that for academics in arts or engineering. By focussing on health academics, the context of this study will ensure that survey findings and proposed solutions are applicable to this specific group of academics with unique challenges to identify problems and find localised solutions. Finally, we focus on solutions as much as identifying and quantifying the problems, by summarising participants’ proposed solutions.

A lack of stable employment and poor mental health in Australia’s health academic workforce may impair advances in these critical life-supporting fields and the delivery of related services [21]. Managing the EMCA experience more appropriately may therefore improve both personal wellbeing and mental health, as well as the productivity and creativity achievable by our sector. Here, we present a descriptive study that explores workplace culture, mental health and wellbeing of health, medical and biomedical EMCAs alongside potential contributors using validated measures.

## Methods

This study was approved by the Medicine and Dentistry Human Ethics Sub-Committee of the University of Melbourne (application 2057562). We adhered to the Strengthening the Reporting of Observational Studies in Epidemiology (STROBE) checklist (Supplementary file 1) [22].

### Participants, settings, and recruitment

Eligible participants were employees in the Faculty of Medicine, Dentistry and Health Sciences (MDHS) at the University of Melbourne, the Faculty of Medicine, Nursing and Health Sciences (FMNHS) and the Faculty of Pharmacy at Monash University. Individuals were asked to self-select, with EMCA defined as an individual with work experience up to ten years full-time equivalent from PhD conferral. Participants provided informed consent through the online survey tool (Qualtrics, Provo, UT) and were recruited via Faculty and EMCA network mailing lists and newsletters between October 2020 and January 2021 (the survey was open for both universities during this time). Incentives were not provided. While it is difficult to estimate the total number of eligible participants, as an indication, the Early Career Academic Network mailing list at MDHS reaches approximately 900 employees, and the EMCA Mailing List for the FMNHS reaches approximately 1100 employees. Both these mailing lists may include employees without a PhD, and not considered an EMCA for the purposes of this study.

### Survey items and validated tools

The survey was developed by a working group including experts in mental health and workplace culture and included several validated tools, including:

- The 16-item *Effort-Reward Imbalance Scale* has separate items on effort (e.g., overtime, constant time pressure, et cetera) as well as rewards (e.g., support, I am treated fairly) [23]. The average effort score is divided by the average reward score, with a total score of 1 or more indicating an individual receiving greater rewards than effort put in, and scores below 1 indicating greater effort than reward. There are two key theoretical models proposed to identify toxic and stressful working conditions: the demand and control model and effort-reward imbalance [24]. The demand-control model posits the importance of psychological demands of work on a person and the degree of control available to the person for required tasks. The demand-control model posits the importance of psychological demands of work on a person and the degree of control available to the person for required tasks [24]. Jobs with high demands and low control cause psychological stress [24]. The effort reward-imbalance emphasises stressful features of work with importance placed on social reciprocity [24]. Social reciprocity defines obligations or tasks in response to adequate rewards that include money, esteem, career opportunities including adequate job security [24].
- The *Short Negative Acts Questionnaire* assesses subjectively experienced exposure to occasional and frequent workplace bullying, using cut-off scores of 12 and 16, respectively [25].
- The 6-item *Ethnic Harassment Experiences* assessed experiences with racism [26].
- The *Sexual Experiences Questionnaire* assesses experiences that fall under four categories [27]. Sexist hostility: gender harassment, discriminatory experiences based on one’s sex (sex discrimination); sexual hostility; harassment experiences that are explicitly sexual (offensive sexual remarks or stories); unwanted sexual attention: sexual behaviours including touching and sexual imposition including assault; and sexual coercion: threats and bribes for sexual favours [28]. Items in the latter 3 categories constitute sexual harassment. Separate items asked participants about experiencing, witnessing, and reporting sexual harassment.
- The *Copenhagen Burnout Inventory* – 7-item Work Related Burnout subscale was used to assess ‘moderate burnout’ (scores of 50 to 74) and ‘high/severe burnout’ (scores of 75–100) [29].
- The *Patient Health Questionnaire 9* (PHQ9) measures overall depression symptoms over the last two weeks. A cut off of >  = 10 can be used to identify people who likely have a Major Depressive Disorder (MDD) [30].
- The *Generalized Anxiety Disorder 7* (GAD-7) measures overall anxiety symptoms over the last two weeks. A cut-off score of 10 represents at least moderate anxiety [31].

Specific questions that apply uniquely to the workplace context and academic misconduct were constructed by the team based on similar workplace surveys [32]. We provided a free text option for participants to provide us with three potential solutions or opportunities for their employers to improve their working conditions. 

### Analyses

All analyses were conducted in Stata v16.0. Participants who exited the survey before completing 20% of the survey items were excluded, as they didn’t contribute data for meaningful analysis. Full case analysis was used. Some items have missing data due to non-completion. Validated tools were scored according to published guidelines. Descriptive analysis was carried out to quantify the prevalence or mean and standard deviation as appropriate. The descriptive analysis was then stratified by subgroups (gender, sexual orientation, race) where considered theoretically important based on literature or expert opinion by the researchers. Poisson regression with robust variance estimator was used to estimate the prevalence ratio (PR) and 95% confidence intervals (95%CI). The goal of this study is not to present causal relationships, therefore only unadjusted prevalence ratios are presented.

The responses to open-ended questions were analysed through a process of content analysis using deductive and inductive coding to identify themes and subthemes by two researchers with qualitative data analysis experience (CHM, DA).

## Results

### Participants

Between 12th November 2020 and 26th January 2021, 320 potential participants started the survey, with 284 participants completing at least 20% of the items. Most participants were female (71.1%), heterosexual (76.4%) and white (71.8%). Most of the participants were within five years of completing their PhD (Table 1).

**Table 1:** Summary of key demographic information for the sample

Variable / response category	Frequency	Percentage
*Age range*		
Up to 30 years	29	10.2%
31—35 years	96	33.8%
36—40 years	75	26.4%
41—45 years	43	15.1%
46—50 years	17	6.0%
Over 50 years	18	6.3%
Prefer not to answer	3	1.1%
*Gender*		
Female	202	71.1%
Male	79	27.8%
Prefer not to say	1	0.4%
*Sexual orientation*		
Heterosexual	217	76.4%
Homosexual	21	7.5%
Bisexual	14	5.0%
Prefer to self-describe	3	1.0%
Prefer not to say	24	8.5%
*Ethnicity*^*a*^		
Caucasian	204	71.8%
Asian	39	13.7%
Indian subcontinent	13	4.6%
Middle Eastern	10	3.5%
South American / Hispanic/ Latino	5	1.8%
Aboriginal / Torres Strait Islander	3	1.1%
Pacific Islander	3	1.1%
African	2	0.7%
Other	8	2.8%
Prefer not to say	9	3.2%
*English as first language*		
No	74	26.1%
Yes	208	73.2%
Missing	2	0.7%
*Permanent resident / citizen*		
No	31	10.9%
Yes	250	88.0%
Missing	3	1.1%
*Carer responsibilities*		
No	133	46.8%
Yes	140	49.3%
Prefer not to say	9	3.2%
Missing	2	0.7%
*Years since completion of research higher degree*		
< 2 years	64	22.5%
2 to < 4 years	56	19.7%
4 to < 6 years	56	19.7%
6 to < 8 years	46	16.2%
8 to < 10 years	32	11.3%
≥ 10 years	26	9.2%
Other	2	0.7%
Missing	2	0.7%
*Employment level*		
Level A, postdoc/ assistant lecturer	79	27.8%
Level B, research fellow/ lecturer	117	41.3%
Level C, senior research fellow/ senior lecturer/	72	25.4%
*Job type*		
Research only	184	64.8%
Research and teaching	66	23.2%
Clinician researcher	19	6.7%
Other	14	4.9%
Missing	1	0.4%

*N* = 284

^a^Multiple responses were possible, therefore total N adds up to more than 284

### Job security and prospects

Of the 280 participants who completed this part of the survey, only 26.8% were on contracts with more than 24 months left or continuing contracts. About a third (*n* = 88, 31.4%) were employed on contracts expiring in less than 6 months, and an additional 54 (19.3%) had less than 12 months remaining. Of these 142 participants with less than 12 months remaining, 34.5% (*n* = 49) did not expect to have their contract renewed.

### Workplace culture and job satisfaction

Of the 281 participants who completed this part of the survey, the majority were somewhat satisfied (47.0%) followed by 26.7% of them being somewhat or very dissatisfied with their work culture. Only 13.5% were very satisfied and 12.8% were neutral regarding the work culture. Through free text responses we identified drivers of dissatisfaction with workplace including “people with stronger power taking credit for accomplishment”, “using and abusing junior researchers as ‘pairs of hands’”, “competition”, “scarcity” and “not valued by the institution”.

Only 11 participants identified as having a disability (3.9%); they scored the extent their university accommodates the needs of individuals with a disability, on a scale of 1–7 (not at all/very much) with an average score of 3.5. Overall, 42.2% of 277 participants indicated they considered leaving academia and an additional 24.9% were unsure. Of those who were unsure or considering leaving, 66 (35.7%) were actively looking or applying for jobs outside academia. The top four reasons for wanting to leave included job insecurity (*n* = 150, 54.7%), lack of funds (*n* = 124, 45.3%), unmanageable workloads (*n* = 89, 32.5%) and lack of career progression (*n* = 86, 31.4%) (multiple responses possible).

Of the 259 participants who completed the Effort-Reward Imbalance scale, 68.0% scored below 1 (more effort than reward), and 32.0% scored 1 or above. Almost all (89.3% of 281) participants reported regularly working overtime (> 40 h per full-time week). About half (51.2%) worked on average 7 or more hours of overtime per week; including 18.2% working more than 12 h of overtime per week. Just over half (51.4%) of the 278 participants agreed that their workplace supports a culture of personal career development. However, only 37.8% felt able to undertake professional development activities relevant to their career aspirations.

### Academic misconduct

Of the 273 participants who completed this part of the survey, 52 (19.0%) reported that they had direct evidence of researchers in their department engaging in any research misconduct in the past 3 years, 204 reported that they had not (74.7%), and 17 preferred not to answer (6.2%). Not adhering to authorship protocols (*n* = 35), and selective reporting/publishing (*n* = 20) were most common, while incidences of plagiarism, falsification and fabrication were less common, each reported by less than 10 participants. It was stated that *“absolutely nothing can be done about authorship due to the power imbalance between junior and senior academics”.* It should be noted that these practices may have occurred at other institutions of prior employment due to the timeframe (previous 3 years)*.*

### Racism

Of the 254 who completed this part of the survey, 57 participants (22.5%) had experienced one or more instances of ethnic harassment/racism (e.g., receiving racist comments or jokes about one’s ethnic group) in the previous 12 months. Of participants who were non-white (28.2% of total sample) 41.6% had experienced ethnic harassment, compared to 14.2% of white participants (2.93 times (95%CI 1.86–4.59) higher prevalence).

### Sexual harassment

Of the 253 participants who completed this part of the survey, 6 (2.4%) indicated ‘yes’ to a survey item asking whether they had experienced sexual harassment at work in the previous year. However, using responses to the Sexual Experiences Questionnaire, 64/253 participants (25.3%) reported having experienced one or more behaviours that constitute sexual harassment (items from the categories sexual hostility, unwanted sexual attention, and sexual coercion, but excluding items from the sexist hostility category) in the workplace in the previous year. Most commonly reported experiences were being told sexual stories or jokes that were offensive and receiving offensive sexual remarks or remarks about one’s appearance, body, or sexual activities. However, 12 participants had experienced unwanted sexual attention (4.7%), and 4 participants reported sexual coercion (1.6%). Sexist hostility (sexism) was reported by 126 (49.8%).

The majority (134 participants, 53.0%) experienced sexism and/or sexual harassment at least once in the previous year. Women experienced this 1.43 times (95% CI 1.05, 1.95) more compared to men (57.8% vs 40.3% respectively); and participants who identified as LGBTIQ + 1.34 time more (95%CI 1.00–1.80) compared to heterosexual participants (65.6% vs 48.7%). Of the 253 participants who responded to the separate item: “Have you witnessed sexual harassment or assault happen to someone else at your current workplace?”, 188 (74.3%) said no, 34 (13.4%) said yes, and the remaining 12.2% said unsure/prefer not to say.

Of 117 participants who identified as having witnessed or experienced any incidence of sexism or sexual harassment, only 24 (20.5%) participants indicated that they had (7, 6.0%) or someone else (17, 14.5%) had reported the incident to management, 48 (41.0%) indicated it had not been reported and 45 (38.5%) were unsure. The level of satisfaction with the management of the incident among the 24 participants was low; 7 (29.2%) indicated they didn’t know how it was managed, 13 (54.2%) said it was managed poorly, 3 (12.5%) said it was managed fairly, and one person (4.2%) indicated it was managed well.

### Bullying

Of the 251 participants who completed this part of the survey, 73 participants (29.1%) experienced occasional bullying, and an additional 44 participants (17.5%) experienced frequent bullying (total *n* = 117, 46.6%). The most frequently reported negative acts included ‘being ignored or excluded’, and ‘someone withholding information which affects your performance’. Participants who identified as LGBTIQ + more often experienced frequent bullying (PR 1.50; 95%CI 1.03, 2.19) compared to heterosexual participants (53.1% vs 35.4% respectively). Participants commented through free text boxes that bullying stemmed from power imbalances, and led to poor work culture, in particular as there was commonly a perceived lack of consequences for bullying.

### Mental wellbeing

Most (54.8%) participants reported work-related burnout, 28.0% scored as having clinically significant symptoms of depression and 21.7% as having clinically significant symptoms of anxiety (Table 2). Regularly working > 12 h of overtime weekly (compared to 0–12 h) and exposure to workplace abuse were all associated with a higher prevalence of burnout, clinically relevant symptoms of depression or anxiety, and suicidal ideation or self-harm (Table 3).

**Table 2:** Mental health of the sample compared to the general population

Variable	EMCAs N (%)	Australian Adults^a^
Burnout^b^	250 (100)	
No burnout	113 (45.2%)	N/A
Moderate burnout	110 (44.0%)	
High/severe burnout	27 (10.8%)	
Depressive symptoms^c^	250 (100)	
No or mild symptoms	180 (72.0%)	72.4%
Clinically significant symptoms	70 (28.0%)	27.6%
Thoughts of being better off dead or of self- harm—At least several days per week^d^	34 (13.6%)	14.6%
Anxiety^e^, N (%)	249 (100)	
No or mild symptoms	195 (78.3%)	79.0%
Clinically significant symptoms	54 (21.7%)	21.0%
Stress, M (SD)	7.28 (2.86)	–

^a^Drawn from a sample of 13,829 Australian adults conducted from 3 April to 2 May 2020

^b^Burnout was measured using The *Copenhagen Burnout Inventory* – 7-item Work Related Burnout subscale to assess ‘moderate burnout’ (scores of 50 to 74) and ‘high/severe burnout’ (scores of 75–100)

^c^Clinically significant depressive symptoms defined as a PHQ-9 score ≥ 10

^d^Variable based on positive response to PHQ-9 item (“thoughts that you would be better off dead or of hurting yourself in some way”)

^e^Clinically significant anxiety defined as a GAD-7 score ≥ 10

**Table 3:** Mental wellbeing by subgroups

	**Moderate or high level of burnout**^**a**^	**Clinically relevant depressive symptoms**^**b**^	**Expressed suicidal ideation or self-harm**^**c**^	**Clinically relevant symptoms of anxiety**^**d**^
	Prevalence ratio; 95% confidence interval; *P*	Prevalence; 95% confidence interval; *P*	Prevalence; 95% confidence interval; *P*	Prevalence; 95% confidence interval; *P*
**Overtime > 12 h per week**	1.52; 1.15, 2.01; *p* = .004	1.81; 1.20, 2.72; *p* = .004	2.81; 1.52, 5.18; *p* = .001	1.90; 1.17, 3.09; *p* = .010
**Exposed to racism**	1.56; 1.21, 2.02; *p* = .001	1.74; 1.16, 2.59; *p* = .007	1.28; 0.63, 2.58; *p* = .496	1.81; 1.12, 2.91; *p* = .016
**Exposed to sexism or sexual harassment**	1.49; 1.14, 1.94; *p* = .003	1.61; 1.06, 2.45; *p* = .027	1.13; 0.60, 2.13; *p* = .700	1.53; 0.93, 2.51; *p* = .091
**Exposed to bullying**	1.50; 117, 1.93; *p* = .001	1.70; 1.14, 2.54; *p* = .010	2.17; 1.14, 4.13; *p* = .018	1.61; 1.00, 2.61; *p* = .050

^a^Burnout was measured using The *Copenhagen Burnout Inventory* – 7-item Work Related Burnout subscale to assess ‘moderate burnout’ (scores of 50 to 74) and ‘high/severe burnout’ (scores of 75–100)

^b^Clinically significant depressive symptoms defined as a PHQ-9 score ≥ 10

^c^Variable based on PHQ-9 item (“thoughts that you would be better off dead or of hurting yourself in some way”)

^d^Clinically significant anxiety defined as a GAD-7 score ≥ 10

Backfill or cover for periods of leave was unavailable to 47.2% (*n* = 109) if they needed time off work and 30.6% (*n* = 68) reported that gradual return to work after leave was not available. Despite the availability of counselling and/or occupational health services, only 7.5% of participants reported using these services.

### Suggestions for improving workplace culture

Practices or initiatives that promote a positive research culture included mentoring for career advice, navigating the academic system, and career progression; networking, collaboration and socialising initiatives; internal grant schemes; EMCA leadership courses and grant management support. Finally, 179 survey participants provided suggestions to improve their workplace experience using free-text responses (Table 4). Themes called for action to: Improve job security and remuneration; Provide career planning support; Invest in people and culture; Manage realistic workloads, reduce burnout, and have clear performance management; and Provide support for grants and funding.

**Table 4:** Thematic analysis of suggestions to improve workplace culture

Theme	Sub themes	Quotes
Improve job security and remuneration	▪ improving job security ▪ pay reflective of qualifications and experience	*Ensure longer contracts and better job security for EMCRs* *Supporting continuity in insecure work environments* *Base level research position for job security* *Better support around the time of contract renewal, ie what financial support you may be eligible for if you don’t get renewed* *Improved wages*
Provide career planning support	▪ career mentoring and coaching ▪ better supporting people from diverse backgrounds and with career interruptions to get promoted ▪ capacity building ▪ clear expectations	*Mandatory Mentorship and career development* *A realistic promotion pathway. The criteria for promotion are just way too hard, particularly if you have more than one circumstance that may affect this e.g. rural location disadvantage, carer responsibilities, teaching (when research metrics are the main metrics looked at.* *Opportunity to develop own career whilst working on someone else’s grant* *Providing realistic information on chances of success/failure in this career early enough so people can make a decision to leave*
Invest in people and culture	▪ promoting positive workplace behaviours and mentoring ▪ a more adequate and transparent process of reporting and managing workplace abuse ▪ improving diversity and inclusion ▪ remove silos through better integration of people into teams, Schools and University	*Appropriately manage supervisors who are performing poorly—have a system of 360 degree feedback that operates all the time, like SETUs [anonymous method for Student Evaluation of Teaching and Units]* *Hold the bullies accountable and listen to the ECRs when they complain* *Create a channel for the EMCR to report issues anonymously* *Provide ways for new (especially international) EMCRs to interact in a social setting* *have more people of colour on ALL panels, committees* *Inclusion—building social connectedness in the team* *Better integration into the school*
Manage realistic workloads, reduce burnout, and have clear performance management	▪ Managing unsustainable workloads ▪ wellbeing support and mental health education for EMCRs and supervisors ▪ collaboratively agreeing on performance expectations ▪ registering overtime, and provide flexi-time and support with leave (backfill)	*Ensure a transparent and reasonable workload for staff* *Achievable, co-developed timelines for outputs* *pay more attention to how many hours EMCRs work and discourage a workaholic culture* *Regular workshops for staff (supervisor) training on mental health—if they don’t know what to do everything else is limited in effect* *More promotion of taking mental health days* *Meaningful strategies to reduce burnout. The 2020 year of COVID disruptions was enormously hard for staff with young families. Yet all we got was 4 days in September to have ‘no email Friday’…woefully inadequate* *Focus on the quality of work instead of pursuing dozens of metrics of quantity.* *Greater emphasis on realistic expectations of EMCRs and realistic career outcomes. I believe many mental health issues arise from a mismatch between unrealistic expectations and reality.*
Provide support for grants and funding	▪ Administrative support for grants ▪ Programs to teach about grant funding ▪ Reduce pressure to apply for category 1 funding ▪ Recognise relative to opportunity including the impact of intersectionality	*increased administrative assistance during grant development* *Research support—although the research office offers support, I wonder if there could be more training into writing a winning grant? Also, there is so much ridiculous admin associated with grants that if there was someone to help we could just focus on the important parts.* *No pressure to apply for Cat 1 schemes* *Funding one’s job is the biggest pressure and i believe leads to poor mental health. No other occupation requires one to find their own salary.* *Options of “relative to opportunity” actually need to be ‘relative to opportunity… not just lip service* *Ability for teaching research staff to be fairly assessed in grants*

## Discussion

Our results reveal a concerning picture requiring urgent attention and intervention; where health, medical and biomedical EMCAs at the two largest and well-resourced, globally recognised universities in Australia experience high levels of job insecurity, coupled with high workload and normalised unpaid overtime. They are commonly exposed to workplace abuse including bullying, harassment, and racism, with half of the respondents reporting work-related burnout and high workloads related to increased symptoms of depression and anxiety.

Our results showed that about half of the participants (47.0%) were satisfied with their workplace culture. This was lower compared to another large survey of Australian EMCA’s (of which half were health and medical sciences EMCAs) which reported 62% job satisfaction in 2019 and 57% in 2022 [8, 10]. Most of our participants (68.0%) reported an effort reward imbalance, i.e. the efforts outweigh the rewards, which is known to cause psychological stress [24]. In comparison, a survey of almost 300 Australian healthcare workers reported 25% had an effort reward imbalance [33]. Job insecurity was ubiquitous, which has previously shown to cause workplace dissatisfaction in EMCAs [8]. Unpaid overtime work was more commonly reported by participants compared to recent reports of Australian workers across other sectors [34]. Experiences with sexism (49.8%) and sexual harassment (25.3%) were also common, despite most of that year being spent in COVID-19 lockdowns limiting in-person interactions. In comparison, 33% of Australian workers experienced sexual harassment in the workplace over 5 years; women, LGBTIQ + , culturally diverse, people with insecure employment, and people with a disability were more commonly affected in this study [35], which is in line with our findings. Our data also shows that most participants were unclear what behaviours constitute sexual harassment, in line with other Australian data [35]. Bullying, experienced by 46.6% of our sample, was far more common than the 8.6% reported in the 2020/2021 Australian Workplace Barometer report (1588 Australian employees across sectors) [9], but similar to another survey of Australian EMCAs [10]. Racism was reported by 22.5% in our study, with non-white people more likely to have experienced ethnic harassment compared to white participants, figures that are very similar to those of The Diversity Council Australia’s Racism at Work report [36].

Workplace abuse included Workplace Health and Safety issues that can lead to serious psychological injury, loss in productivity (beyond the victim), staff turnover, reputation loss and workers’ compensation claims [35, 37]. Following a 2016 report detailing sexual harassment experienced by 21% of students across 39 Australian Universities [38], both Universities involved in our research have made commitments to reduce sexual harm on campus including improved reporting avenues, and transparency on outcomes of complaints. However, our data indicate that reporting these experiences to management is still uncommon, and the handling of these reports was deemed very unsatisfactory. Another Australian survey of EMCAs recently also reported that few people (22.4%) were willing to report workplace abuse due to low confidence in leadership to take action and concerns about repercussions [10]. As far as we are aware, there are no major initiatives to specifically and systematically combat bullying or racism in academic workplaces. Academic misconduct, which may occur in high-stress environments with high competition, and considered a serious offence, was reported by 19% of participants. This was lower than the 35% of participants who reported questionable research practices in a recent Australian-wide survey, but their definition was perhaps interpreted differently [8].

Burnout was reported by 54.8% of participants, higher than reports of burnout rates among Australian midwives (51%) [39], and psychologists (27.8%) around the same time [40]. A UK study of academics also reported high burnout rates, and higher than average rates of mental health problems [4]. Rates of clinically significant symptoms of depression, anxiety, and suicidal ideation in our sample were comparable to those seen in the Australian adult population during the COVID-19 pandemic [41]. However, mental health issues in EMCAs working > 12 h of overtime weekly greatly exceeded these rates [41], in line with a study of graduate students using similar measures of mental health [42]. This could be indicative of mental health issues manifesting early in the academic career track when job insecurity is ubiquitous, and the pressure to perform and obtain funding is very high. Exposure to workplace abuse was also associated with poorer mental health status; further adding weight to the urgent need to address structural factors that enable these issues. Rather than workplace policies and training, by far the greatest predictors of the occurrence of workplace abuse are organisational factors [35, 37]. Some of these factors that are highly relevant to universities include: hierarchical organisations which cultivate isolation, over-representation of men in senior leadership, poor work-life balance, disproportionate drive towards results and excellence rather than wellbeing of staff, power imbalance, and a history of protecting the reputation of perpetrators [35, 37]. It is the responsibility of the leadership in our universities to combat these determinants of poor workplace culture. Provision of a safe and secure work environment for employees is an employer’s responsibility, which may require a structural approach addressing organisational factors [35, 37].

Solutions offered by our participants may go some way in improving working conditions, including improved job security, providing career planning, meaningful investment in people, diversity and workplace culture, strategies to reduce burn-out, and managing realistic workloads. However, the organisational factors that make this industry particularly vulnerable to workplace abuse might require a structural rethink of how universities are organised, how they value their people and measure their performance [43].

### Implications

Our findings, from data collected at two of Australia’s largest universities, show that EMCAs working conditions are worse compared to other Australian sectors. Urgent action is needed if universities are to ensure a safe and inclusive work culture and to attract and retain a diversity of EMCAs in health and medical research [8]. Other countries such as the United Kingdom have already acknowledged workplace culture problems and job insecurities faced by this workforce and have proposed action plans to attract and retain staff [4, 44]. Based on our findings and recommendations, addressing high workloads, job insecurity, and workplace abuse in Australian universities are viewed as top priorities. The current ‘survival of the fittest’ culture is likely impacting the diversity of the academic workforce, hindering highly qualified and talented individuals to progress and reach their potential in academia. Although our study design did not allow for causal analyses, addressing these workplace issues may go some ways to reduce the high prevalence of burnout, and symptoms of depression and anxiety in EMCAs [24]. Subgroups of the EMCA community are disproportionately affected by these issues, including more junior staff, women, and other historically disadvantaged groups [24]. Well-designed cohort studies that collect extensive covariate data on the key causal questions from our study are warranted to quantify the causal effects of negative experiences on mental health on this population group and clarify experiences of smaller subgroups that may be more exposed to negative working conditions. This will help quantify the avoidable poor mental health burden by intervening on specific exposures within highly regarded academic institutions.

### Strengths and limitations

This was a grassroots effort delivered by EMCAs. The major strength of this study is the use of a range of validated instruments, which sets the basis for comparisons with studies performed in other sectors, other regions, as well as future studies in this cohort. The survey data was collected while there were some COVID-19 pandemic restrictions in place, and therefore EMCAs with high workloads, caregiving responsibilities, and/or severe mental distress may not have participated in the survey. Conversely, individuals who were highly motivated to express their dissatisfaction may be more likely to participate. Therefore, our data may over- or underrepresent some groups, and over- or underestimate the occurrence or severity of issues such as overtime and sexual harassment. Subgroup analyses were hindered by small numbers; e.g. no one identified as gender non-binary, preventing us from accurately quantifying their experiences. We are unable to verify whether our sample is representative of the EMCA workforce at Australian universities overall, as universities do not collect staff data on time since PhD conferral considering career interruptions [8]. Further, the two universities included in this study are both located in Melbourne, and both are members of the Group of Eight (G08), considered world-leading research-intensive universities. Therefore, our sample may not be generalisable to other, smaller universities or those located outside Melbourne. However, we also note that having this specific sample allows the relevant stakeholders to make evidence informed interventions to address the challenges faced by EMCAs across the two large universities. Finally, given the cross-sectional nature of the survey, causality or temporality should not be inferred and was not an aim.

### Supplementary Information

**Supplementary Material 1. **

## Data Availability

The data are not publicly available for sharing. A request can be made to the corresponding author and will require institutional ethics approval.
